# Interaction Control to Synchronize Non-synchronizable Networks

**DOI:** 10.1038/srep37142

**Published:** 2016-11-17

**Authors:** Malte Schröder, Sagar Chakraborty, Dirk Witthaut, Jan Nagler, Marc Timme

**Affiliations:** 1 Network Dynamics, Max Planck Institute for Dynamics and Self-Organization (MPIDS), 37077 Göttingen, Germany; 2 Department of Physics, Indian Institute of Technology Kanpur, U.P. 208016, India; 3 Forschungszentrum Jülich, Institute for Energy and Climate Research - Systems Analysis and Technology Evaluation (IEK-STE), 52428 Jülich, Germany; 4 Institute for Theoretical Physics, University of Cologne, 50937 Köln, Germany; 5 Computational Physics, IfB, ETH Zurich, Wolfgang-Pauli-Strasse 27, 8093 Zurich, Switzerland; 6 Department of Physics, Technical University of Darmstadt, 64289 Darmstadt, Germany

## Abstract

Synchronization constitutes one of the most fundamental collective dynamics across networked systems and often underlies their function. Whether a system may synchronize depends on the internal unit dynamics as well as the topology and strength of their interactions. For chaotic units with certain interaction topologies synchronization might be impossible across all interaction strengths, meaning that these networks are non-synchronizable. Here we propose the concept of *interaction control*, generalizing transient uncoupling, to induce desired collective dynamics in complex networks and apply it to synchronize even such non-synchronizable systems. After highlighting that non-synchronizability prevails for a wide range of networks of arbitrary size, we explain how a simple binary control may localize interactions in state space and thereby synchronize networks. Intriguingly, localizing interactions by a fixed control scheme enables stable synchronization across all connected networks regardless of topological constraints. Interaction control may thus ease the design of desired collective dynamics even without knowledge of the networks’ exact interaction topology and consequently have implications for biological and self-organizing technical systems.

One of the simplest and most common types of collective dynamics of a networked system is synchrony, the state in which all units behave identically^1^,^2^. Synchrony emerges, and is often essential, in natural and artificial systems alike, e.g. in the dynamics of circadian oscillators and neural circuits as well as in communication networks and power grids^3^,^4^,^5^,^6^,^7^,^8^,^9^,^10^. More than 25 years ago, Pecora and Carroll^11^,^12^,^13^ uncovered that even chaotic units may synchronize; under certain conditions they coordinate their dynamics even though individually the units generate dynamics that are sensitive to small variations in the initial conditions.

The types of chaotic units jointly with their interaction topology and strength determine whether synchronization is possible at all^14^. Some combinations of system types and interaction topologies do not enable synchronization of the units for *any* coupling strength, rendering those systems non-synchronizable^13^. Yet, several technical systems demand synchronization of their units^10^,^15^,^16^,^17^,^18^,^19^, requiring generic methods to achieve synchronization, ideally despite such obstacles. In fact, chaos synchronization has attracted a broad range of applications from secure communication to new paradigms of network analysis^17^,^20^,^21^,^22^,^23^,^24^.

In this article, we investigate how a simple control of network interactions guarantees reliable synchronization independent of the specific interaction topology. We first highlight that a wide range of systems with sparse connectivity are non-synchronizable, even if they exhibit at least indirect connections (paths) between any two units. We then systematically extend a method of transient uncoupling that has been studied for two coupled oscillators^25^ to propose a general scheme of *interaction control* applicable to any network. We show that localizing the interactions among the units to small regions of state space not only extends the synchronization range but newly creates synchrony, even for non-synchronizable networks. We further show that interaction control in fact enables synchronization regardless of the underlying interaction topology. The proposed scheme of interaction control leaves the system entirely non-interacting in most of state space, potentially saving interaction costs. Interaction control may thus help establishing collective dynamical states desired for network function in a simple and efficient way.

## Results

### Problem setting

Consider networks of *N* units with dynamics given by


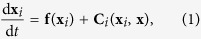


where 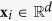 is the state of unit *i*, **f**(**x**_*i*_) describes the internal dynamics and **C**_*i*_(**x**_*i*_, **x**) represents the pairwise interactions between the unit’s state variable **x**_*i*_ with the full network’s state 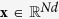. The interactions are given by


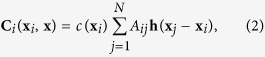


where *A*_*ij*_ ∈ {0, 1} denotes the adjacency matrix of the undirected interaction network and **h** is the interaction function. For the control scheme introduced below, we write *c*(**x**_*i*_) to be a general control function that localizes interactions in state space (see below). For a system without control we have constant *c*(**x**_*i*_) = *α*.

For the numerical examples presented throughout this article we consider the units as Rössler systems^26^ given by 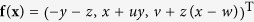 with parameters *u* = *v* = 0.2 and *w* = 5.7 and diffusive coupling with 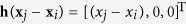 for **x** = (*x*, *y*, *z*)^T^. Interaction control is equally applicable in other settings, specifically for Rössler oscillators with different parameters, networks of other chaotic units and systems with various types of limited observability (see Supplementary Information for details).

### Prevalence of non-synchronizable networks

**Figure 1:**
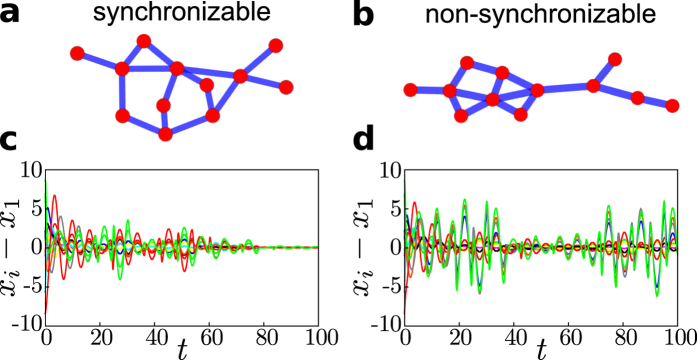
Synchronizable or not? Similar networks may exhibit different synchronization properties. (**a**,**b**) Two networks, despite having the same number of units, identical units, identical coupling strength and identical degree sequences exhibit different synchronizability (here for *N* = 12 coupled Rössler oscillators with identical parameters (see text), and identical coupling strength *α* = 0.5). (**c**) Network (**a**) enables stable synchronization. (**d**) For network (**b**) the synchronized state is unstable. More generally, no choice of *α* results in stable synchronization: in this sense, the network is entirely non-synchronizable.

**Figure 2:**
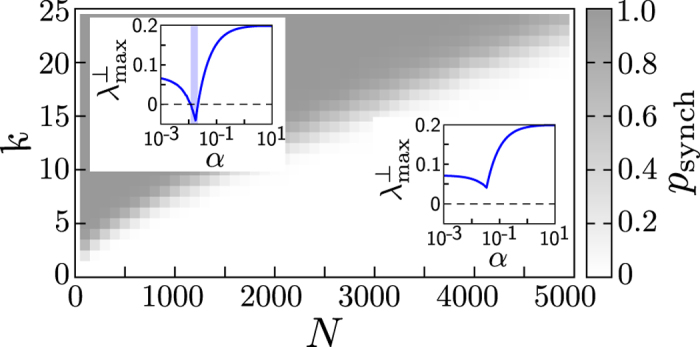
Prevalence of non-synchronizable networks. Synchronizability of ensembles of Barabási-Albert networks^49^ of *N* units with *M* = *Nk* links. The main panel shows the probability *p*_synch_ that these networks are synchronizable (measured as the fraction of 100 networks that enable stable synchronization for some value of the coupling strength *α*). The panel shows a clear transition to non-synchronizable networks with increasing sparseness (decreasing *k*). Insets: examples of the largest transverse Lyapunov exponent as a function of the coupling strength *α* for networks of *N* = 1000 units. Top left: synchronizable network (*k* = 16); the range of coupling strengths *α* enabling synchronization is shaded. Bottom right: Non-synchronizable network (*k* = 4).

Synchronizability of such networks of chaotic units depends on the interaction topology. Trivially, if the network is not connected synchronization is impossible. Yet, even connected networks exhibiting (at least) indirect interaction paths among every pair of units may be non-synchronizable, compare also^27^,^28^. Indeed, whereas some networks may be synchronizable, similar networks with similar statistics of their topologies are non-synchronizable: Fig. 1 illustrates topology and dynamics of two networks, where one is synchronizable and the other is not, despite both having identical dynamical units and identical degree sequence. More generally, we highlight that a large fraction of sparse networks with heterogeneous degree sequence is indeed non-synchronizable (Fig. 2).

### Control to localize interactions

The most obvious way to change the synchronizability of a non-synchronizable network is modifying the network topology such that the synchronized state becomes stable, an approach followed previously^29^,^30^. However, changing the topology is often costly, if not impossible, in particular if the exact network topology is unknown. Can stable synchronization be achieved for these non-synchronizable networks at all?

Let us control the interactions to a small, local part of state space and now take *c*(**x**_*i*_) in Eq. (2) to be a binary switch as in ref. 25: the control function then regulates whether the units are coupled at strength *c*(**x**_*i*_) = *α*, if the local state is in some small region of state space where ||**x**_*i*_ − **s**|| < *r* (for some offset point **s**), or whether the units do not interact, *c*(**x**_*i*_) = 0 otherwise. In the limit of *r* → ∞, the units interact for all **x**_*i*_ in state space such that *c*(**x**_*i*_) ≡ *α* and we recover the original network of coupled chaotic units.

For small *r* the control strongly localizes the interactions (e.g., in the following examples with *r* = 2.75 interaction is active only about 5% of the time), thereby vastly reducing the information exchanged across the network. For communication systems, for instance, where information exchange comes with energetic or other costs, such interaction control might reduce these costs by limiting the interactions. At the same time, localizing the interactions in this way stabilizes synchrony for a range of choices of **s** and *r*. In the following examples, we employ **s** ≈ (−8.7, 2.3, 0.01)^T^ (see Supplementary Information “Choice of the coupling region” and Fig. S1) and systematically vary the localization radius *r* of the coupling as well as the coupling strength *α*.

### Enabling synchronization by interaction control

**Figure 3:**
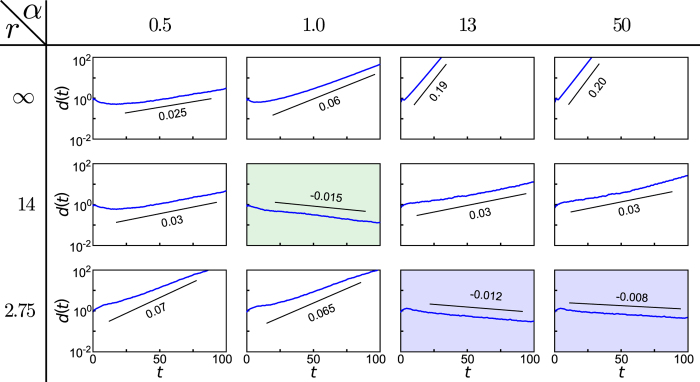
Interaction control to synchronize non-synchronizable networks. Panels display the average convergence or divergence of trajectories for the non-synchronizable interaction network displayed in Fig. 1(b). Averages are taken over *R* = 500 initial conditions randomly drawn from boxes of linear size 0.01 around points drawn randomly on the attractor. Each panel displays the relative average divergence of the states of the units 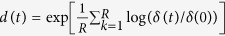 where 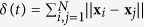. The black lines illustrate the scaling expected from the maximum transverse Lyapunov exponent. Without control (*r* = ∞) the network is non-synchronizable, independent of the coupling strength *α*. For moderate control, the system is synchronizable (negative exponent, highlighted by green shading) for some intermediate range of *α*. For strong control where interactions are highly localized, stable synchrony prevails at sufficiently large coupling strengths (highlighted by blue shading).

Intriguingly, we find that interaction control may reliably enable synchronization of networks if the interactions become strongly localized. In particular, synchronization is achieved even for systems that are non-synchronizable without control. We first illustrate the effects of interaction control for the small non-synchronizable network displayed in Fig. 1(b) in dependence of coupling strength *α* and localization radius *r*, see Fig. 3. For small *r* (highly localized coupling), synchronization becomes stable as long as the coupling strength is sufficiently large. For moderate *r*, synchronization is still possible, but only in some interval of coupling strengths. Without control (*r* = ∞), however, the network is entirely non-synchronizable.

**Figure 4:**
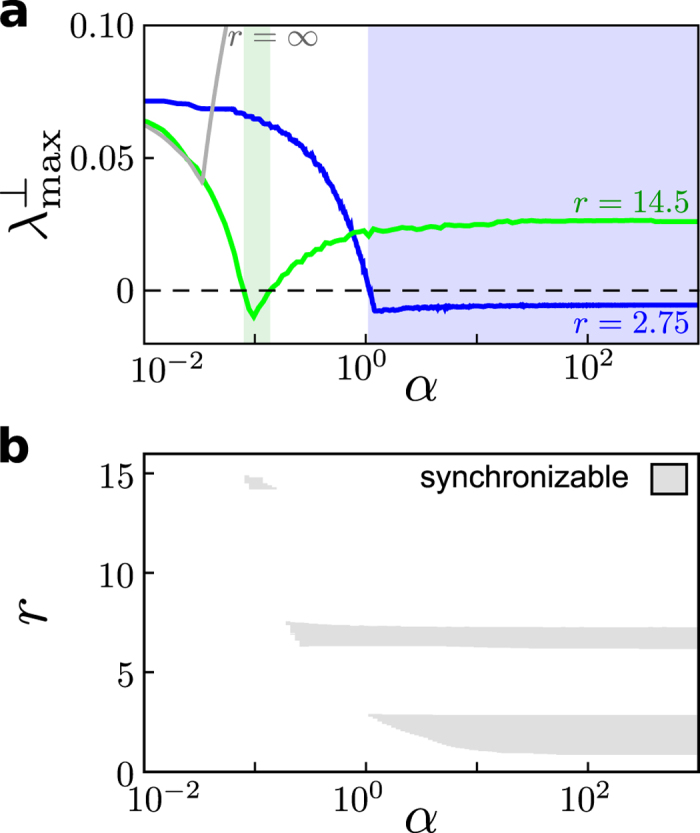
Control generically enables synchronization for large networks. Localized interactions induce stable synchronization even in large networks with arbitrary topology. Panel (a) shows the largest transverse Lyapunov exponent for a non-synchronizable Barabási-Albert network of *N* = 1000 units and *k* = 4 without control (light gray line, compare Fig. 2) and with interaction control. Moderately localized interactions (control parameter *r* = 14.5) enable stable synchronization in a small range of coupling strengths only (shaded in green). Stronger localization (*r* = 2.75) enables stable synchronization for all sufficiently large coupling strengths (shaded in blue). Panel (b) illustrates the synchronizability for all combinations of coupling strength *α* and localization radius *r*.

**Figure 5:**
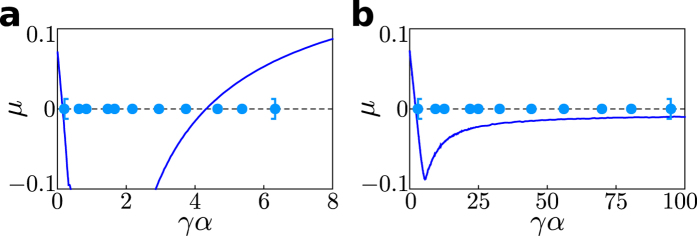
Stable synchronization for arbitrary network topologies. Master stability function *μ* for real values *γα*, where *γ* are the eigenvalues of the Laplacian of the interaction network and *α* is the coupling strength (see also Supplementary Information “Extension of the master stability formalism”). The light blue points mark *γα* for the eigenvalues of the non-synchronizable network displayed in Fig. 1(b). (**a**) Without control the network is non-synchronizable because some transverse modes are unstable regardless of the choice of *α* (shown here for *α* = 1). (**b**) With interaction control (*r* = 2.75) all transverse modes are stable (negative 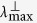) if the coupling strength is large enough (*α* = 15), since the master stability function is negative for large *γα*. Similarly, interaction control can be used to synchronize any connected, undirected network independent of its topology, since for sufficiently large coupling strengths all transverse modes will become stable.

To understand how interaction control is successful in enabling synchronization, consider the following intuitive argument: compare the local Lyapunov exponents (expansion or contraction) of the system with and without coupling. In some regions of state space the coupled system will be less expanding (more contracting) than the uncoupled system while in other regions the coupled system is more expanding (less contracting). Intuitively, applying interaction control and activating coupling only at the former, more contracting regions will lead to overall stronger contraction and will thus be beneficial for synchronization. This is the basic mechanism of interaction control. Note, however, that this argument is only approximate as it neglects the impact of interaction control on the local Lyapunov exponents: for instance, activating coupling only in one region, *A* or *B*, might be beneficial for synchronization, whereas activating coupling in both, *A* and *B*, might destabilize the synchronized state due to the effect of coupling in *A* on the effectiveness of coupling in *B* (see also Supplementary Information “Choice of the coupling region” and Fig. S1). Nevertheless, the general mechanism is applicable for a wide range of network structures and chaotic systems:

In fact, the qualitative behavior is robust for larger networks and, intriguingly, generalizes to all connected network topologies: Fig. 4 illustrates the typical characteristics for a network of *N* = 1000 units taken from the regime of non-synchronizability displayed in Fig. 2, now with interaction control localizing the coupling up to a parameter *r*. Systematically varying both *r* and *α* shows a common pattern: (i) For large *r*, i.e. without control or only weak localization, the system remains non-synchronizable (ii) For moderate *r*, the system becomes synchronizable for a finite interval of coupling strengths. (iii) For some sufficiently small *r*, even non-synchronizable networks become synchronizable for an infinite range of coupling strengths. Combined with the fact that every finite network has a finite and thus bounded spectrum the theory of master stability^14^ implies that interaction control may enable stable synchronization for *all* connected network topologies by strongly localizing where the units interact: for sufficiently large coupling strengths all eigenvalues of any finite, undirected graph fall within the range of negative transverse Lyapunov exponents, see Fig. 5.

Furthermore, interaction control can enable synchronization not only across network topologies but is successful for a range of different dynamical units and under various observability conditions. For instance, for different parameters in the chaotic regime of the Rössler oscillator we find qualitatively the same results to those presented above [see Supplementary Information “Rössler oscillator for different parameters” and Fig. S2 (a,b)]. Additionally, we find similar effectiveness of interaction control for different other dynamical systems, e.g., Lorenz^31^ and Chen^32^ oscillators (see Supplementary Information “Lorenz System”, “Chen System” and Fig. S3 and S4 respectively). Moreover, interaction control is applicable in networks with limited observability or limited controllability: All of the above examples already demonstrate successful interaction control with only one of the dynamical variables (for example only *x*) observed for each unit. We also find that interaction control can enable synchronization when measurements are possible at only a few discrete points in time [see Supplementary Information “Rössler oscillator for different parameters” and Fig. S2(c)]. Finally, we considered interaction control in networks where a fraction of units is not observable and thus not directly controllable. Depending on the interaction topology of the entire network, all units may become synchronizable through interaction control under some conditions on the coupling strengths. Generally, the controlled part of the network stays or even becomes synchronizable in the presence of interaction control, irrespective of its topology (see Supplementary Information “Partially controlled networks” and Fig. S5).

## Conclusion

Many networks are non-synchronizable for various types of coupled units and across all interaction strengths because synchronizability is intrinsically limited by the topology of the interaction network. Here we propose interaction control to synchronize arbitrary networks, even if they are entirely non-synchronizable without control. Generalizing the idea of transient uncoupling previously suggested for two coupled oscillators^25^ to arbitrary networks is thereby generically successful and operates by localizing interactions in state space. The interaction control scheme requires no changes to the network topology and exploits only a binary switch to strongly localize interactions to a small region in state space. As it works across all network topologies, the topology of any given network even need not be known.

Previous studies discussing time or state dependent uncoupling enhanced the stability of the synchronized state and extended synchronizability of systems that are already synchronizable^25^,^33^,^34^,^35^. Related works aimed at enabling synchronization in non-synchronizable networks focused on topological constraints and permanent changes to the network topology^36^,^37^,^38^ or adaptive coupling strengths requiring permanently active interactions and detailed control over the coupling of the individual units^39^,^40^. In contrast, in this article we demonstrate how interaction control may synchronize previously non-synchronizable networks. Specifically, interaction control induces a *qualitative* rather than a quantitative change of the synchronizability interval that cannot be explained by extending existing ranges of synchronizability or modifying effective coupling strengths. As we report, non-synchronizability prevails among sparse networks with heterogeneous degree sequence, emphasizing the range of systems for which interaction control may be valuable.

Intuitively, interaction control increases synchronizability by disabling coupling in regions of state space where the trajectories of the coupled system diverge more than those of the uncoupled system. Thus, interaction control enables synchronization with little information transmission between the units, thereby providing a potentially efficient control for engineered systems where interaction generates costs in terms of energy or other resources^33^,^41^, for example for communication with chaos synchronization^17^,^42^. At the same time, interaction control can be successfully applied to induce synchronization even in systems with limited observability. For instance access may only be possible to some of the dynamical variables of each node, measurements at discrete points in time or in the presence of unobservable and thus uncontrollable units (see Supplementary Information), opening up potential perspectives, e.g., also for natural and synthetic biological systems^43^,^44^. Following the intuitive mechanism, from a general dynamical systems perspective interaction control might be applicable to any collective state that exhibits instabilities due to coupling among variables. Specifically, this includes potentially inducing different collective dynamics, for instance phase synchronization, or enabling coordinated dynamics also for delayed or pulsed interactions^36^,^37^,^45^.

In summary, interaction control offers a generic control scheme for collectively coordinated networks. Although requiring interaction only in a small region of state space, interaction control enables synchronization in all connected networks independent of their specific topology, even if the network would normally be non-synchronizable. Generally, interaction control may functionally help beyond enabling synchrony, for instance to create consensus among interacting agents^46^. Interaction control may thus offer a complementary network control method^47^,^48^ and thereby a valuable paradigm for enabling a number of different collective dynamical phenomena in a range of networked systems.

## Additional Information

**How to cite this article**: Schröder, M. *et al.* Interaction Control to Synchronize Non-synchronizable Networks. *Sci. Rep.* **6**, 37142; doi: 10.1038/srep37142 (2016).

**Publisher’s note:** Springer Nature remains neutral with regard to jurisdictional claims in published maps and institutional affiliations.

## Supplementary Material

Supplementary Information
