# Supplementary material for: Interaction Control to Synchronize Non-synchronizable Networks
Source: Sci Rep. 2016 Nov 17;6:37142. doi: 10.1038/srep37142 (PMC5112558; doi:10.1038/srep37142)
Supplement: Supplementary Information [file srep37142-s1.pdf]

Supplementary Material
accompanying the manuscript
Interaction Control to Synchronize Non-synchronizable Networks

Malte Schröder, Sagar Chakraborty, Dirk Witthaut, Jan Nagler, Marc Timme

In the main manuscript, we discuss how interaction control creates synchronizability for networks of coupled chaotic units. Specifically, we demonstrated that interaction control enables to synchronize networks that are non-synchronizable without control, irrespective of their network topology. Here, we first discuss the choice of the offset point $\mathbf{s}$ used for the examples in the main manuscript. Second, we formally extend the master stability formalism[1] to include interaction control. Third, we illustrate the universality of this approach by applying interaction control to networks of Rössler systems at different parameters, to networks of Lorenz and to networks of Chen systems with qualitatively the same results as those presented in the main manuscript. Finally, we consider three aspects of interaction control in systems with limited observability, specifically, when we only have access to a single variable of each unit, to measurements at discrete points in time and in the presence of unobservable (and thus uncontrollable) units in the network.

Throughout this supplement we use the same notation as in the main manuscript

$$\frac{\mathrm{d}\mathbf{x}_i}{\mathrm{d}t} = \mathbf{f}(\mathbf{x}_i) + \mathbf{C}_i(\mathbf{x}_i, \mathbf{x}), \tag{1}$$

where $\mathbf{x}_i \in \mathbb{R}^d$ is the state of unit i, $\mathbf{f}(\mathbf{x}_i)$ describes the internal dynamics and $\mathbf{C}_i(\mathbf{x}_i, \mathbf{x})$ represents the pairwise interactions between local state variable $\mathbf{x}_i$ and the remaining network's state $\mathbf{x} \in \mathbb{R}^{Nd}$ for a network of N units. Again, the interactions are defined as

$$\mathbf{C}_i(\mathbf{x}_i, \mathbf{x}) = c(\mathbf{x}_i) \sum_{j=1}^{N} A_{ij} \mathbf{h}(\mathbf{x}_j - \mathbf{x}_i), \tag{2}$$

where $A_{ij} \in \{0, 1\}$ denotes the adjacency matrix of the undirected interaction network, $\mathbf{h}$ is the interaction function and $c(\mathbf{x}_i)$ is a general control function that localizes interactions in state space.

Supplementary Note 1

Choice of the coupling region.

In the examples in the main manuscript we employed interaction control via

$$c(\mathbf{x}_i) = \begin{cases} \alpha & \text{if} \quad ||\mathbf{x}-\mathbf{s}|| < r \\ 0 & \text{else} \end{cases}, \tag{3}$$

where $\mathbf{s} \approx (-8.7, 2.8, 0.01)^{\mathrm{T}}$ and r controls the size of the coupling region. Here we explain how to choose $\mathbf{s}$. To find suitable parameters for interaction control we computed the stability of the synchronized state of two coupled Rössler systems for various choices of potential offset points $\mathbf{s}'$ and a suitable distance $r(\mathbf{s}')$. We chose $R = 10000$ points randomly from the attractor (invariant measure) as potential offset points. For each point we calculate $r(\mathbf{s}')$ such that $c(\mathbf{x}_i) = \alpha$ for a fraction of 5% of points on the invariant measure. With these parameters and $\alpha = 5$ we calculated the maximum transverse Lyapunov exponent $\lambda_{\mathrm{max}}^{\perp}$. The point $\mathbf{s}$ for which the maximum transverse Lyapunov exponent is minimal is chosen as the offset point. Results of the simulations are shown in Fig. S1. Depending on choice of $\mathbf{s}$, interaction control will be more or less efficient. If one effectively optimizes the function $c(\mathbf{x}_i)$ to increase stability one might expect even better results, allowing for example stable synchronization with minimal coupling effort.

A faster way to determine a feasible, though probably less efficient, coupling region can be understood by considering a simple argument that qualitatively explains how interaction control works: comparing the local Lyapunov exponents for the uncoupled and coupled system provides a measure of how effective coupling is at any given point in state space. A suitable coupling region restricts coupling to efficient points and, more importantly, disables it at points where coupling is detrimental to synchronization. One would then naturally expect more stable synchronization. This method requires only derivatives of the individual dynamics $\mathbf{f}(\mathbf{x}_i)$ which are either known or can be estimated from measurements and will provide a feasible coupling region for general systems that is expected to enhance synchronizability. Note, however, that this method uses only local indicators but ignores global effects, such as coupling at one point changing the effectiveness of coupling at another point. Thus, while this method might serve as an efficient way to determine a feasible coupling region, it is not guaranteed to result in an efficient one.

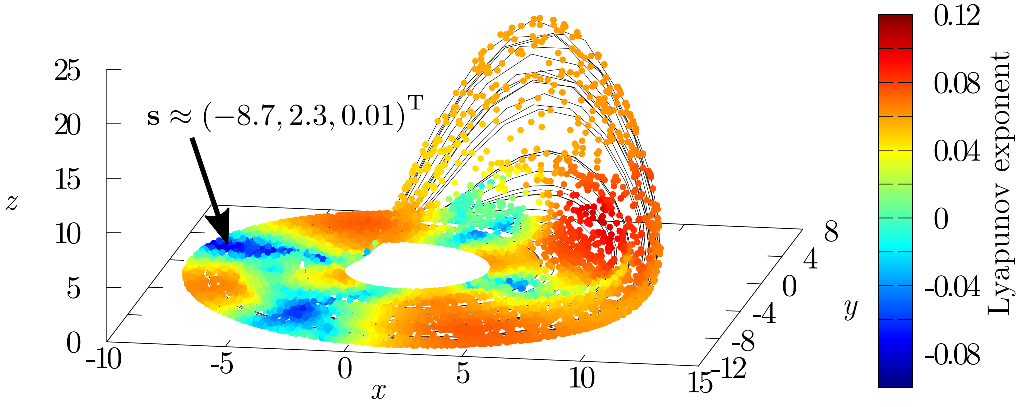

Figure S1. Largest transverse Lyapunov exponent of two coupled Rössler systems, figure adapted from[2]. Simulations were done with parameters $\mathbf{s}'$ (marked by the location of the points), $r(\mathbf{s}')$ such that $c(\mathbf{x}_i) = \alpha$ for a fraction of 5% of points on the invariant measure and $\alpha = 5$ (see text). The color indicates the resulting largest transverse Lyapunov exponent. The most stable synchronized state (minimum largest transverse Lyapunov exponent) is achieved for $\mathbf{s} \approx (-8.7, 2.8, 0.01)^{\mathrm{T}}$ (indicated by the arrow) and marks our choice for the offset point.

Supplementary Note 2

Extension of the master stability formalism. Here we derive the extension of the master stability function formalism introduced in[1] for interaction control. We assume the same dynamics as described in Eq. 1.

The equation describing our network of N coupled, identical units with dynamical variables $\mathbf{x}_i \in \mathbb{R}^d$ in d dimensions can then be written as

$$\frac{\mathrm{d}\mathbf{x}}{\mathrm{d}t} = \tilde{\mathbf{F}}(\mathbf{x}) + \tilde{\mathbf{C}}(\mathbf{x})(\mathbf{G}\otimes\mathbf{H})\,\mathbf{x}, \tag{4}$$

where $\mathbf{x} \in \mathbb{R}^{dN}$ describes the vector of states of all oscillators and we write $\tilde{\mathbf{F}}(\mathbf{x}) = (\mathbf{f}(\mathbf{x}_1), \mathbf{f}(\mathbf{x}_2), \dots)^{\mathsf{T}}$ [similarly for $\tilde{\mathbf{C}}(\mathbf{x})$ combining the individual $c(\mathbf{x}_i)$]. The coupling is defined by $\mathbf{G} \in \mathbb{R}^{N\times N}$ describing the Laplacian of the coupling network, $\mathbf{H} \in \mathbb{R}^{d\times d}$ defining the coupling between the coordinates and $\mathbf{G}\otimes\mathbf{H}$ representing the direct product. As an example, two bidirectionally coupled units with coupling between the x-coordinates would be described by

$$G = \begin{pmatrix} -1 & 1 \\ 1 & -1 \end{pmatrix}, \qquad H = \begin{pmatrix} 1 & 0 & 0 \\ 0 & 0 & 0 \\ 0 & 0 & 0 \end{pmatrix}. \tag{5}$$

The variational equations around the synchronous orbit $\mathbf{x}_S$ then read

$$\begin{aligned} \frac{\mathrm{d}(\delta\mathbf{x})}{\mathrm{d}t} &= \mathrm{D}\tilde{\mathbf{F}}(\mathbf{x}_S)\delta\mathbf{x} + \tilde{\mathbf{C}}(\mathbf{x}_S)(\mathbf{G}\otimes\mathbf{H})\,\delta\mathbf{x} + \left[\mathrm{D}\tilde{\mathbf{C}}(\mathbf{x}_S)\,\delta\mathbf{x}\right](\mathbf{G}\otimes\mathbf{H})\,\mathbf{x}_S \\ &= \left[\mathrm{D}\tilde{\mathbf{F}}(\mathbf{x}_S) + \tilde{\mathbf{C}}(\mathbf{x}_S)(\mathbf{G}\otimes\mathbf{H})\right]\delta\mathbf{x}, \end{aligned} \tag{6}$$

since $(\mathbf{G}\otimes\mathbf{H})\,\mathbf{x}_S = 0$. These equations can then be described in terms of eigenvectors of the coupling network

$$\frac{\mathrm{d}\xi_k}{\mathrm{d}t} = \left[\mathrm{D}\mathbf{f}(\mathbf{x}_S) + \gamma_k c(\mathbf{x}_S)\,\mathbf{H}\right]\xi_k \tag{7}$$

for $k \in \{1, 2 \dots N\}$ where γ_k are the eigenvalues of $\mathbf{G}$, the $\xi_k \in \mathbb{R}^d$ are small variations with respect to the synchronous orbit and $\mathrm{D}\mathbf{f}(\mathbf{x}_S)$ is the Jacobian matrix of a single unit. It is thus sufficient to study the master stability function $\mu(\gamma\alpha)$, defined as the largest Lyapunov exponent of the system

$$\frac{\mathrm{d}\xi}{\mathrm{d}t} = \left[\mathrm{D}\mathbf{f}(\mathbf{x}_S) + \gamma c(\mathbf{x}_S)\,\mathbf{H}\right]\xi, \tag{8}$$

to determine the stability of arbitrary networks with interaction control.

In the following we use $c(\mathbf{x}_i) \in \{0, \alpha\}$ as in the main manuscript. We compute the master stability function for real values of the parameter $\gamma\alpha$ (undirected networks) both with and without control. To illustrate the effect of interaction control we show results for eigenvalues of a non-synchronizable network, illustrating how it becomes synchronizable with interaction control.

Supplementary Note 3

Rössler oscillator for different parameters. We again consider a network of Rössler units[3] with dynamics [Eq. (1)] given by

$$\mathbf{f}(\mathbf{x}) = \begin{pmatrix} -(y+z) \\ x+uy \\ v+z(x-w) \end{pmatrix} \tag{9}$$

and coupled only in the x-coordinate, i.e., $\mathbf{h}(\mathbf{x}_j - \mathbf{x}_i) = [(x_j - x_i), 0, 0]^{\mathrm{T}}$ for $\mathbf{x} = (x, y, z)^{\mathrm{T}}$. However, we use a different set of parameters: $u = v = 0.1$, $w = 14$. Here, we choose the control function for interaction control as

$$c(\mathbf{x}_i) = \begin{cases} \alpha & \text{if} \quad |x_i - x^*| < d \\ 0 & \text{else} \end{cases}, \tag{10}$$

where $x^* = 1.325$ is the center of the attractor in x-direction, i.e. we localize control to a box of width $2d$ in x-direction. We illustrate results for the master stability function without and with control for $d = 10$ in Fig. S2 (a,b). Note that both the coupling as well as the coupling region only depend on the x-coordinate, thus showing that interaction control can be successfully applied without access to the y and z variable of the system. Furthermore, in Fig. S2 (c), we assumed limited observability in the sense that measurements of the unit's states are only possible at discrete time points with only about five measurements per full oscillation. Consequently, we can only adjust the control function at these times: at the time of a measurement, the state of the coupling is fixed as active ($c = \alpha$) or inactive ($c = 0$) depending on the current state of the unit for a time Δt_{meas} until the next measurement. Note that the coupling *input* [i.e. $\mathbf{x}_j - \mathbf{x}_i$] is still continuous in time. As in the main manuscript, all networks become synchronizable regardless of their specific topology due to the interaction control, even if they were non-synchronizable without control.

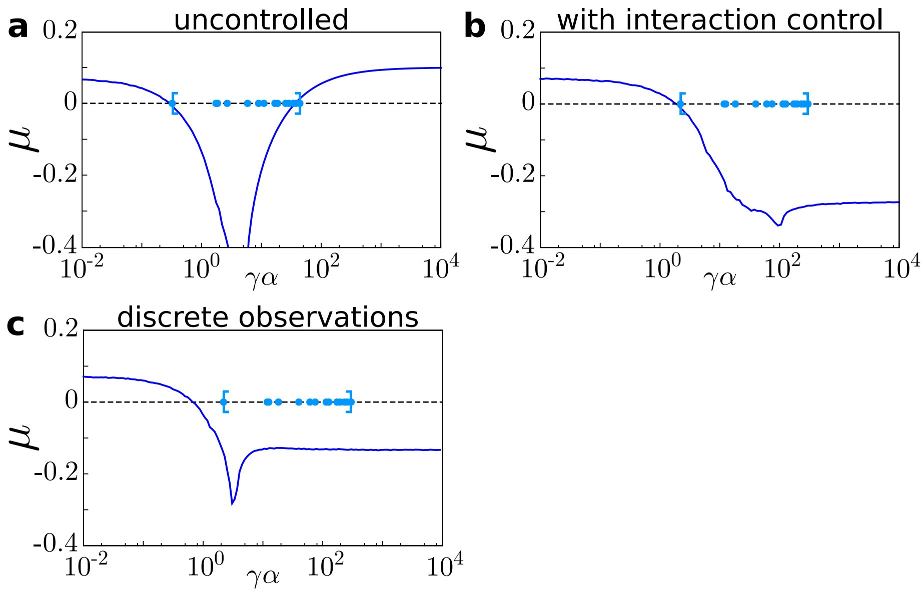

Figure S2. Master stability function of coupled Rössler units for real values $\gamma\alpha$, where γ are the eigenvalues of the Laplacian of the coupling network and α is the coupling strength. The light blue points illustrate eigenvalues of a non-synchronizable network with $\alpha = 2$ [panel (a)] and $\alpha = 10$ [panel (b)]. a) Without control the network is non-synchronizable, some transverse modes are unstable. b) With interaction control (Eq. (10), $d = 10$) all transverse modes are stable if the coupling strength is sufficiently large, since the master stability function is negative for all sufficiently large $\gamma\alpha$. Similarly, interaction control can be used to synchronize any undirected network independent of its topology, since for large coupling strengths all transverse modes will be stable. c) Even with limited observability, i.e., only about five measurements per full oscillation ($\Delta t_{\mathrm{meas}} = 1$), interaction control is still successful in enabling stable synchronization regardless of network topology.

Supplementary Note 5

Lorenz system. As another example we consider Lorenz units[4] with dynamics [Eq. (1)] given by

$$\mathbf{f}(\mathbf{x}) = \begin{pmatrix} \sigma\,(y-x) \\ x\,(\rho-z)-y \\ xy-\beta z \end{pmatrix} \tag{11}$$

with $\sigma = 10$, $\rho = 28$ and $\beta = 8/3$ and coupled with $\mathbf{h}(\mathbf{x}_j - \mathbf{x}_i) = [0, 0, (z_j - z_i)]^{\mathrm{T}}$. Interaction control is realized with

$$c(\mathbf{x}_i) = \begin{cases} \alpha & \text{if} \quad |z_i - z^*| < d \\ 0 & \text{else} \end{cases}, \tag{12}$$

where we chose $z^* = 25$ approximately in the center of the attractor in z-direction. We illustrate results for the master stability function without and with control for $d = 1$ in Fig. S3. While all networks of coupled Lorenz oscillators are synchronizable for very large coupling strengths, interaction control both decreases the coupling strength necessary to induce stable synchronization and increases the stability of the synchronized state, enhancing synchronizability of all networks.

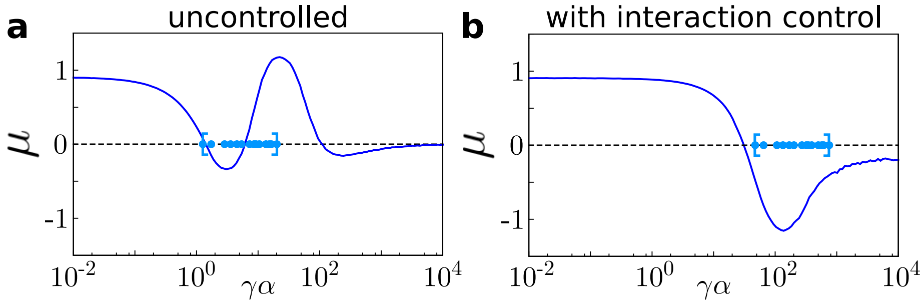

Figure S3. Master stability function of coupled Lorenz units for real values $\gamma\alpha$, where γ are the eigenvalues of the Laplacian of the coupling network and α is the coupling strength. The light blue points illustrate eigenvalues for an example network with $\alpha = 3$ [panel (a)] and $\alpha = 100$ [panel (b)]. a) Without control the synchronized state is unstable since some transverse modes are unstable. Synchronization would be (weakly) stable only for very large coupling strengths. b) With interaction control (Eq. (12), $d = 1$) all transverse modes are stable if the coupling strength is sufficiently large, since the master stability function is negative for all sufficiently large $\gamma\alpha$ and the stability of the synchronized state is enhanced. Similarly, interaction control can be used to synchronize any undirected network independent of its topology, since for large coupling strengths all transverse modes will become stable.

Supplementary Note 6

Chen system. As a final example we consider Chen units[5] with dynamics [Eq. (1)] given by

$$\mathbf{f}(\mathbf{x}) = \begin{pmatrix} u\,(y-x) \\ (w-u)\,x - zx + wy \\ xy - vz \end{pmatrix} \tag{13}$$

with $u = 35$, $v = 3$, $w = 28$ and coupled with $\mathbf{h}(\mathbf{x}_j - \mathbf{x}_i) = [0, 0, (z_j - z_i)]^{\mathrm{T}}$. Interaction control is realized with

$$c(\mathbf{x}_i) = \begin{cases} \alpha & \text{if} \quad |z_i - z^*| < d \\ 0 & \text{else} \end{cases}, \tag{14}$$

where we chose $z^* = 26.5$ approximately in the center of the attractor in z-direction. We illustrate results for the master stability function without and with control for $d = 5$ in Fig. S4. As in the main manuscript, all networks become synchronizable regardless of their specific topology with interaction control, even if they were non-synchronizable without control.

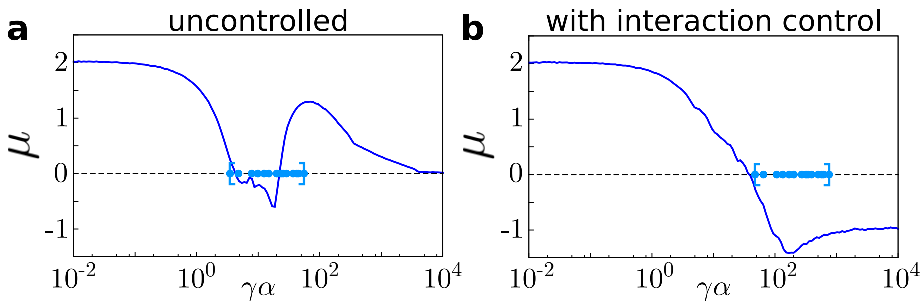

Figure S4. Master stability function of coupled Chen units for real values $\gamma\alpha$, where γ are the eigenvalues of the Laplacian of the coupling network and α is the coupling strength. The light blue points illustrate eigenvalues of a non-synchronizable network with $\alpha = 8$ [panel (a)] and $\alpha = 100$ [panel (b)]. a) Without interaction control the network is non-synchronizable, some transverse modes are unstable. b) With interaction control (Eq. (14), $d = 5$) all transverse modes are stable if the coupling strength is sufficiently large, since the master stability function is negative for all sufficiently large $\gamma\alpha$. Similarly, interaction control can be used to synchronize any undirected network independent of its topology, since for large coupling strengths all transverse modes will become stable.

Supplementary Note 7

Partially controlled networks. We consider a network where some units are not observable and thus not affected by interaction control, these uncontrolled nodes are instead continuously coupled to their neighbors $[c(\mathbf{x}_i) = \alpha]$. In such partially controlled networks, success of the method, by construction, depends on the set of controlled units and the network structure. The general effect can be readily understood considering the simplified case of a single uncontrolled unit: we split the network into two disjoint sets, a (connected) set of controlled units A and a set of the single uncontrolled unit B, as sketched in Fig. S5(a). Consider now the two parts separately: the controlled part A will synchronize as any other network under the effect of interaction control for coupling strength $\alpha \geq \alpha_{\text{min,A}}$. Considering input from B to A as a small outside perturbation, interaction control still enables stable synchronization of the controlled part A.

Assuming A is synchronized, unit B receives input in form of the synchronized state from all its connections to A. Synchronization will typically be stable only in a finite range of coupling strengths, $\alpha \in [\alpha_{\text{min,B}}, \alpha_{\text{max,B}}]$ (e.g. for Rössler or Chen systems). Thus, synchronizability of the complete network A and B is either possible in a finite interval of coupling strengths if $\alpha_{\text{min,A}} < \alpha_{\text{max,B}}$ or synchronization is only stable in part A of the network if $\alpha_{\text{min,A}} \geq \alpha_{\text{max,B}}$.

For larger sets of uncontrolled units the structure of the individual sets and their interaction becomes more important. The general idea, however, holds: both parts of the network must be synchronizable for the same coupling strength in order to allow synchronization of the complete network [illustrated in Fig. S5(b-e)].

In summary, success of interaction control in partially controlled networks depends on the network structure. Whereas synchronization of the whole network possible under the (necessary) condition $\alpha_{\text{min,A}} < \alpha_{\text{max,B}}$, the controlled part A of the network will always be synchronizable for large enough coupling strengths (disregarding the perturbation by part B). This potentially enables selective control over specific parts of a given network as long as the outside disturbance of the uncontrolled units is not too large.

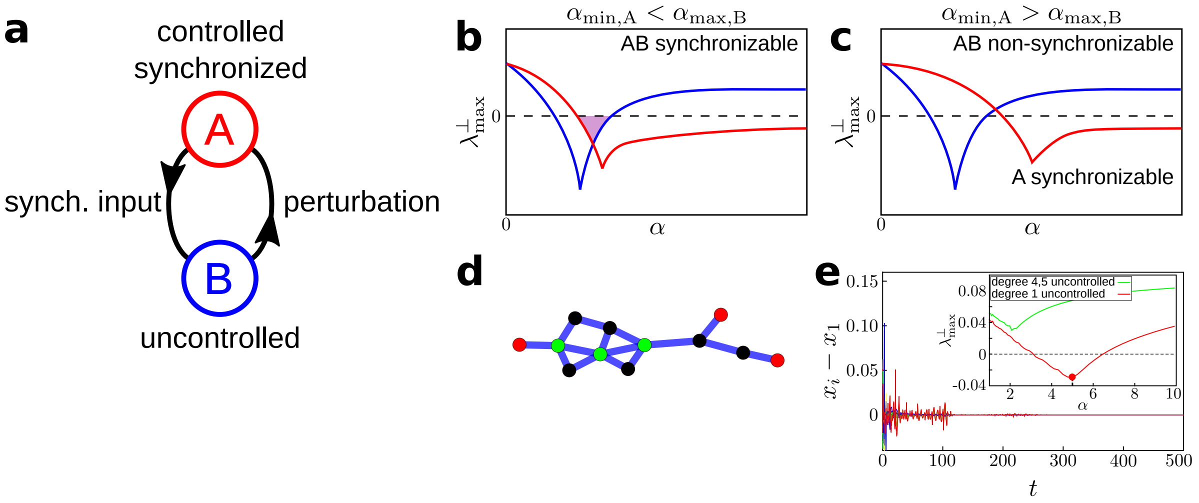

Figure S5. Panel (a) shows a schematic of a partially controlled network: we consider two sets of units, controlled (A) and uncontrolled (B). Panel (b) and (c) show a sketch of the maximum transverse Lyapunov exponent for the two parts A and B in two different cases. While interaction control guarantees synchronizability of the controlled part A as long as it is connected (disregarding the perturbation from B), synchronization of part B is typically only possible in a small range of coupling strengths, if at all. Consequently, synchronization of the complete network is only possible in the the finite range of coupling strengths illustrated in panel (c) if both A and B are synchronizable for the same coupling strength. Panel (d) shows an example of a partially controlled network of Rössler units (see above: "Rössler oscillator for different parameters"). Two sets of uncontrolled nodes are marked in green and red, the corresponding maximum Lyapunov exponent is shown in the inset of panel (e). If nodes with large degree [green in panel (d)] are uncontrolled, complete synchronization of the network is not possible and the system diverges. For another set of uncontrolled nodes [red in panel (d)] synchronization is possible in a finite range of coupling strengths. Panel (e) shows one set of trajectories for the second case with $\alpha = 5$, the differences between the units disappear and all units synchronize.

References

1. Pecora, L. M. & Carroll, T. L. Master stability functions for synchronized coupled systems. *Phys. Rev. Lett.* **80**, 2109–2112 (1998).

2. Schröder, M., Mannattil, M., Dutta, D., Chakraborty, S. & Timme, M. Transient uncoupling induces synchronization. *Phys. Rev. Lett.* **115**, 054101 (2015).

3. Rössler, O. E. An equation for continuous chaos. *Phys. Lett. A* **57**, 397–398 (1976).

4. Lorenz, E. N. Deterministic nonperiodic flow. *J. Atmos. Sci.* **20**, 130–141 (1963).

5. Chen, G. & Ueta, T. Yet another chaotic attractor. *Int. J. Bifur. Chaos* **09**, 1465–1466 (1999).
